# Ivy: A Multi-modal Verification Tool for Distributed Algorithms

**DOI:** 10.1007/978-3-030-53291-8_12

**Published:** 2020-06-16

**Authors:** Kenneth L. McMillan, Oded Padon

**Affiliations:** 8 Microsoft Research Lab, Redmond, WA USA; 9 University of Southern California, Los Angeles, CA USA; 10 Microsoft Research, Redmond, USA; 11 Stanford University, Stanford, USA

## Abstract

Ivy is a multi-modal verification tool for correct design and implementation of distributed protocols and algorithms, supporting modular specification, implementation and proof. Ivy supports proving safety and liveness properties of parameterized and infinite-state systems via three modes: deductive verification using an SMT solver, abstraction and model checking, and manual proofs using natural deduction. It supports light-weight formal methods via compositional specification-based testing and bounded model checking. Ivy can extract executable distributed programs by translation to efficient C++ code. It is designed to support decidable automated reasoning, to improve proof stability and to provide transparency in the case of proof failures. For this purpose, it presents concrete finite counterexamples, automatically audits proofs for decidability of verification conditions, and provides modular hiding of theories.

## Introduction

Ivy is an open-source [16] multi-modal verification tool for correct design and implementation of distributed algorithms, supporting modular specification, implementation and proof. The motivating principles of Ivy are *predictability*, *stability* and *transparency*. That is, automated proof steps should provide complexity bounds, should be insensitive to small perturbations, and when they fail should provide actionable feedback. To the extent consistent with these principles, Ivy aims to maximize expressiveness and proof automation, and thus to achieve a high level of user productivity in designing, implementing and proving programs. A major goal of Ivy is to support *decidable reasoning*. That is, automated proof should be restricted to logical fragments for which the tool is a decision procedure. This greatly improves the stability of automated provers, which otherwise rely on fragile heuristics to avoid divergence [28]. This is important for the maintenance of large proofs, to prevent small changes from creating unpredictable proof failures. Moreover, on decidable problems, provers fail transparently by providing true counterexamples, which greatly simplifies the iterative development of proofs. Ivy supports the decomposition of proofs to decidable theories by the use of modular abstraction.

**Fig. 1.**
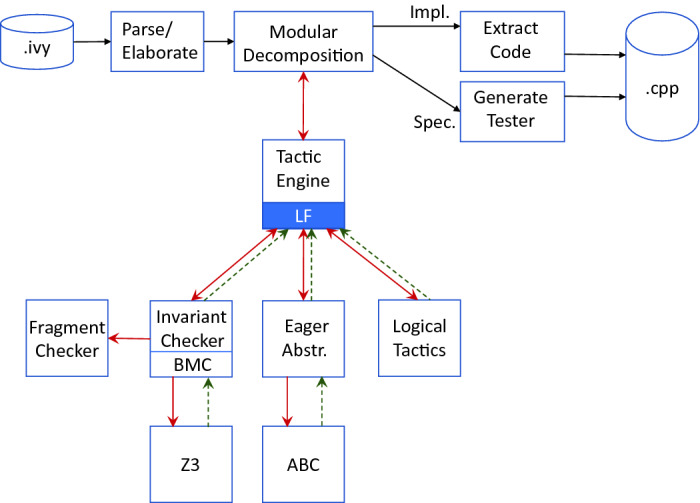
Ivy architecture, showing flow between major components. Red, solid arrows represent flow of proof goals and assumptions. Green, dashed arrows represent flow of proofs and/or counterexamples. Not shown is VC generator, shared between Invariant Checking/BMC and Eager Abstraction components. (Color figure online)

The architecture of Ivy is depicted in Fig. 1. The figure shows the major components of the tool and the information flow between them. Ivy provides a language (also called “Ivy”) for the modular description of distributed programs, along with their specifications and proofs (see Sect. 2). Ivy is a synchronous, reactive programming language [3], meaning that the program only executes actions in response to input from its environment, and these actions appear to execute atomically. From an Ivy program, the tool can extract an asynchronous, distributed implementation. A program is made up of reactive modules [1], each having a temporal assume/guarantee-style specification. After parsing of this description and elaboration of templates, the program is decomposed into its component modules, each with associated assumptions and proof obligations, according to a system of proof rules for circular assume/guarantee reasoning (see Sect. 2.1).

These proof obligations are passed on to the tactics engine (see Sect. 3). This engine orchestrates the use of various built-in proof tactics, including decidable invariant checking with an SMT solver (Sect. 3.1), model checking with eager abstraction [19] (Sect. 3.2), liveness proof by translation to safety (Sect. 3.3) and logical deduction rules (Sect. 3.4). Each tactic works by reducing a given proof goal to a (possibly empty) set of sub-goals, from which the original goal can be proved. Combined with modular reasoning, the tactics engine makes it possible to use a variety of proof approaches and proof automation tools in constructing a proof.

Ivy extracts executable distributed programs by translation to C++ (see Sect. 5). From the specifications of a module, Ivy can also generate a modular randomized specification-based tester [7] (see Sect. 4.1). This also makes it possible to test infrastructure not written in Ivy (including hardware) against Ivy specifications.

### Related Work

Ivy can be thought of as a hybrid between program verification tools such as ESC-Java [11] and Dafny [14], based on the Floyd/Hoare approach, compositional model checking tools, such as Mocha [2] and Cadence SMV [17] and proof assistants based on the LCF model, such as Isabelle [26] or Coq [4]. Compared to program verification tools that support only procedure modularity, Ivy provides a richer form of specification that allows complete hiding of internal state, and provides architectural support for decidable reasoning (see Sect. 2.1). Compared to compositional tools, Ivy integrates a richer variety of reasoning techniques (see Sect. 3). Compared to proof assistants, Ivy provides domain-specific support for decidable proof automation, supporting a greater degree of proof automation [28]. On the other hand, Ivy relies on a vastly larger trusted computing base than typical proof assistants. Moreover, Ivy has no mechanism of reflection, and thus cannot be used for meta-reasoning about programs and program transformations. In principle, all the techniques in Ivy could be integrated into a tool such as Isabelle or Coq but the effort would be large. A less foundational tool such as Ivy makes it possible to rapidly experiment with new proof and proof automation strategies. Compared to all of these tools, Ivy differs in providing native support for extracting distributed programs, and specification-based testing. A related tool, mypyvy, focuses on more powerful invariant inference techniques, but lacks the other features of Ivy [10, 29].

## A Modular Language for Decidable Reasoning

**Fig. 2.**
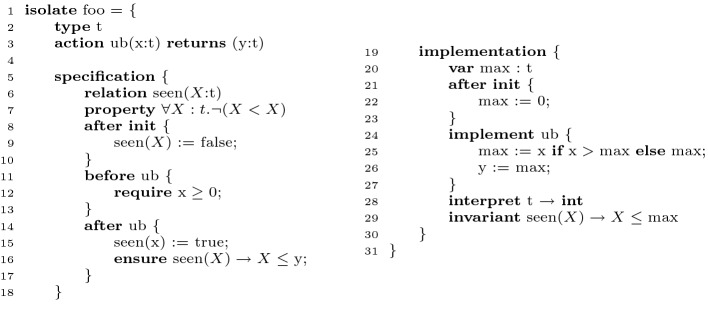
Example of an Ivy isolate.

The primary design goal of Ivy’s language is to support decidable reasoning while maximizing expressiveness and performance. Figure 2 is an example of the basic unit of verification in Ivy, called an *isolate*. An isolate is a reactive module that hides internal state and provides a temporal (that is, stateful) specification of its interface. An isolate has named traits that include types, properties, variables and actions. It is divided into a *specification* part and an *implementation* part. The figure shows an example of a simple module that inputs a sequence of numbers and outputs an upper bound on the numbers received thus far.

**Types, Variables and Actions.** The native datatypes in Ivy include just the Boolean type, uninterpreted types, records (structs) over datatypes, and pure first-order functions. In the figure, line 2 declares an uninterpreted type *t*. Line 6 declares a state variable ‘seen’ holding a predicate over *t*. This variable is initialized at line 9. This assigns ‘seen(*X*)’ to be the function that returns false for all values of *X*.

Procedures in Ivy are called *actions* and may have side effects on variables. Parameters are passed by value and there are no references. This greatly simplifies modular reasoning (see Sect. 2.1) and also allows for aggressive compiler optimizations due to the absence of aliasing (see Sect. 5).

In the figure, line 3 declares an action ‘ub’ that takes an input *x* of type *t* and outputs *y* of type *t*. Its implementation is given at lines 24 to 27. It updates a state variable ‘max’ holding the maximum value received thus far, and returns this value by assigning it to the output variable *y*.

### Modularity and Decidability

The specification part of the isolate (lines 5 to 18) consists of *ghost* variables and code that are *visible* outside the isolate. The implementation part (lines 19 to 30) consists of *real* variables and code that are *invisible* outside the module. At line 15 the ghost predicate ‘seen’ is updated to reflect the fact that value *x* has been seen as an input. Specification code contains assume/guarantee specifications in terms of require and ensure statements. For example, line 12 represents an assumption that input values are non-negative. Line 16 represents a guarantee that output values will be an upper bound on all seen values.

Ghost and real code are kept syntactically separate in Ivy. The specification code is interleaved with the implementation code using the directives ‘before’ (line 11) and ‘after’ (line 14). Thus, in the figure, the ‘require’ statement acts as a precondition, while the ‘ensure’ statement acts as a postcondition. The implementation code is not allowed to side effect any externally visible state, so it is sound to erase (or ‘slice’) this code when verifying other modules. Other modules see only the ghost code, which provides an abstract model of the isolate. Similarly, when extracting executable code, it is safe to erase the ghost code (which must be proven to be terminating). This makes it possible, for example, to provide a pure, functional specification of a module interface, even though internally it has state.

Theories can also be hidden inside modules. For example, the implementation of our example interprets the type *t* as the integers (line 28). For verification purposes, this instantiates the theory of Peano arithmetic for type *t*. This theory is used *only* to prove correctness of the isolate, and is invisible to other isolates. The theory can be used to prove properties (such as the irreflexivity property at line 7) that provide an abstraction of the type externally. The ability to hide theories behind abstractions provides an important strategy for keeping proof obligations decidable.

An isolate with no implementation part (that is, a “ghost” module) can act as an abstract model of a protocol. Using Ivy’s modular rules, an abstract model can be *refined* to an implementation, using properties of the abstract model as lemmas. In addition to simplifying the proof, abstract models provide another useful strategy to hide functions, properties or theories that break decidability. This approach, in combination with theory hiding, was used to verify implementations of distributed consensus protocols [28]. Modularity provides the primary means in Ivy of keeping the automated reasoning decidable.

## Verification Tactics

Ivy provides a range of automated tactics for discharging proof goals that are selected for their relatively predictable and stable performance, and for the ability to fail transparently.

### Invariant Checking with SMT

The default tactic for proving safety properties is proof by inductive invariant, using the SMT solver Z3 [21]. For example, in Fig. 2, the guarantee at line 16 is proved using the auxiliary inductive invariant at line 29. The invariant relates the hidden implementation state variable ‘max’ with the visible specification state variable ‘seen’. An invariant is a property that is required to hold only between executions of actions of the isolate. That is, actions may temporarily violate an invariant, but must re-establish it before terminating. The VC (verification condition) for the isolate holds if all invariants are established by the intializers and preserved by the interface actions, and if the invariant implies that no assertion in the code fails. These conditions are verified modulo the visible theories.

Before attempting to prove the VC, the invariance tactic sends it to the *fragment checker*, which determines whether the VC is in a logical fragment called FAU [12] for which Z3 is a decision procedure. If the VC is not in FAU, Ivy provides an explanation to the user, by pointing to formulas that create a *function cycle* or that violate rules for the use of quantifiers and interpreted operators of the visible theories. A function cycle is a cycle in a graph whose vertices are types and whose edges are functions (including Skolem functions). This transparent mode of failure helps the user to reorganize the proof to keep the VC’s in the decidable fragment.

If a VC in the decidable fragment is false, Z3 fails transparently, producing a true finite counter-model, which is in turn translated into an execution trace that violates an invariant or guarantee. Ivy provides a graphical interactive tool to help the user in strengthening invariants [25] based on counterexamples. If the VC is valid, the tactic discharges the proof goal, returning the empty set of subgoals.

### Eager Abstraction and Model Checking

An alternative tactic to prove safety properties is model checking with eager abstraction [19]. This technique allows parameterized and infinite-state systems to be verified with a finite-state model checker. The tactic first propositionally strengthens the symbolic transition relation by adding instances of axioms of the logic and theories, or of proved properties. It then propositionally abstracts the transition relation by converting the atomic predicates to Boolean variables. The resulting finite-state abstraction is verified by the ABC model checker [8]. If the property is false, the user is presented with an abstract counterexample expressed in terms of the truth values of the atomic propositions. The user may refine the abstraction by adding instantiation terms or auxiliary invariants. In [19] it was shown that this technique can reduce the burden of constructing auxiliary invariants, simplifying the overall proof of distributed protocols. As an example, the isolate of Fig. 2 can be proved without the auxiliary invariant. With eager abstraction, one need not be concerned with function cycles, but on the other hand, diagnosing abstract counterexamples can be challenging.

This approach is consistent with Ivy’s philosophy of using stable and transparent automation, since the finite-state model checker has a single-exponential upper complexity bound and terminates with a proof or a counterexample. This is in contrast to more powerful proof engines such as Horn solvers [6] that suffer from unpredictable divergence. In practice, although eager abstraction is not fully automated, it can handle problems that are substantially beyond the capabilities of current Horn solvers.

### Liveness-to-Safety Transformation

Ivy supports proofs of temporal properties, e.g., liveness properties, via a liveness-to-safety transformation. Temporal properties are specified in first-order linear temporal logic (FO-LTL). The liveness-to-safety tactic reduces a temporal proof goal into a safety proof goal, which can then be proven using an inductive invariant. For finite-state or parameterized systems, any temporal property can be proven by showing the absence of fair cycles, which is a safety property [27]. For infinite-state systems such an argument is not sound, and Ivy implements *dynamic abstraction* which generalizes the notion of fair cycles to infinite-state systems in a sound and powerful way [23, 24]. With dynamic abstraction, Ivy’s liveness-to-safety tactic supports temporal proofs of infinite-state systems, including both distributed systems with infinite-state per process and systems with *unbounded parallelism*, where new processes can be dynamically created so an infinite trace may involve infinite set of processes.

The liveness-to-safety tactic fits within Ivy’s philosophy of using decidable reasoning. The more standard way of proving liveness properties is to use ranking functions, but for distributed systems, the required rankings often involve cardinalities of sets defined via first-order formulas, resulting in verification conditions that fall outside FAU and other decidable fragments. In contrast, the transformation to safety based on fair cycles and dynamic abstraction results in verification conditions which are often in the FAU fragment. Furthermore, since the temporal proof is transformed to a safety verification problem, it is possible to leverage for liveness proofs all the tactics and mechanisms that Ivy contains for safety verification.

When the liveness-to-safety tactic is applied, Ivy constructs a symbolic *cycle detection transition system*, which tracks fairness constraints and includes a *shadow* or *saved copy* of the state variables, similar to [5]. For finite-state or parameterized systems, it is enough to show that it is not possible to revisit the saved state while satisfying all fairness constraints. This can be shown by an inductive invariant, and Ivy contains special syntax for writing the invariant of the cycle detection system (e.g., to access the saved copy of state variables). For infinite-state systems, Ivy’s cycle detection system includes dynamic abstraction, and invariants may also refer to the state of the abstraction [23].

**Fig. 3.**
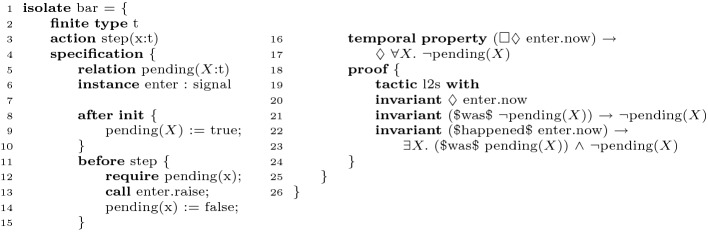
Example of an Ivy isolate with a temporal property.

Figure 3 shows an example of a simple liveness proof of an abstract model in Ivy. The type *t* (line 2) is declared as finite, which means it is sound to use a fair cycle argument without dynamic abstraction. The specification state of the system consists of a single unary relation, $$\text {pending}$$, which is initialized to true for all values of type *t*. The step action (line 11) removes a single value from the $$\text {pending}$$ relation. This can model, e.g., execution of tasks from a finite pool of pending tasks. The temporal property that we prove (line 16) is that if step is called infinitely often, then eventually nothing is pending. At line 13, we detect the call by raising a flag $$\text {enter.now}$$. The proof applies the liveness-to-safety (l2s) tactic (line 19), and supplies inductive invariants for the cycle detection system. The special operators $was$ and $happened$ are used to refer to the saved state, and the fairness constraints, respectively. The crux of the invariant is that after $$\text {enter.now}$$ has happened, there is some element which was pending in the saved state and is not pending anymore, showing that the system has no fair cycle.

### Logical Tactics

**Fig. 4.**
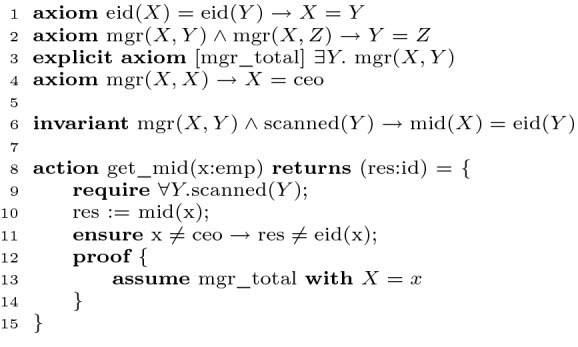
Example of manual quantifier instantiation with a tactic

Though most of a proof in Ivy is done with the above automated proof tactics, there are occasional situations in which a small amount of detailed manually-guided proof is needed, or is preferable to restructuring the proof. For this purpose, Ivy provides logical proof tactics that can be applied to properties, invariants or code assertions, either to complete the proof or to reduce it to subgoals that can be discharged by the automated tactics. A simple example is shown in Fig. 4. Here, $\text {mgr}(X,Y)$ indicates that the manager of employee *X* is *Y* and $\text {eid}(X)$ is the employee id of *X*. We assume that employee ids are unique, each employee has exactly one manager and that only the CEO is her own manager (lines 1 to 4). Action get_mid(*x*) returns the id of the manager of employee *x*. For this purpose, a procedure (not shown) scans the employees *m* and sets $\text {mid}(x) = \text {eid}(m)$ for each *x* managed by *m*, establishing the invariant at line 6. Action get_mid(*x*) requires that all employees have been scanned and ensures that the return value is not the id of *x*, unless *x* is the CEO.

Axiom mgr_total states that for all employees there exists a manager (the universal quantifier on *X* is implicit). Ivy complains that this quantifier alternation puts the VC outside the decidable fragment. We can solve this with a manual quantifier instantiation. We first tag the axiom *explicit*, meaning that it is not used by the default tactic. We then apply the tactic ‘assume’ (line 13) to instantiate this axiom for $X=x$. The resulting assumption $\exists Y. \text {mgr}(x,Y)$ has no alternation. The modified proof goal is discharged by the default tactic using Z3. Ivy’s proof engine is based on the $\lambda \varPi$ calculus [13] and a deterministic second-order matching algorithm [30]. The Ivy standard library uses this framework to define proof rules for natural deduction, similarly to Isabelle/FOL [26]. Logical tactics also make it possible to perform theory reasoning outside the decidable fragment, for example, applying the Peano induction axiom.

## Light-Weight Formal Methods

### Compositional Specification-Based Testing

Before attempting a formal proof that an isolate satisfies its specification, it is useful to debug it using testing. For this purpose, Ivy provides compositional specification-based testing. The testers that Ivy produces generate randomized input sequences for an isolate that satisfy its assumptions and check the outputs against the isolate’s guarantees. This is similar in principle to specification-based testing tools such as QuickCheck [9], but is reactive and compositional. Compositionality provides a kind of completeness for unit testing. That is, if a system fails its specification, then there is a local test of some component that fails. Unlike QuickCheck, Ivy does not require the user to provide generators for datatypes, instead relying on SMT solving for this purpose. Ivy can also be used to generate specification-based tests for hardware or software systems not written in Ivy. For example, it has been used to find bugs in memory hierarchy components for RISC-V processors [18], and the QUIC secure Internet transport protocol [20].

### Bounded and Finite-State Model Checking

For debugging, Ivy supports bounded model checking. This is decidable if the VC’s are in the decidable fragment. It also allows uninterpreted types to be finitely instantiated, allowing under-approximate model checking in the style of TLC [31].

## Extracting Efficient Executable Code

*Compilation.* The implementation part of an Ivy program can be extracted as executable code in C++. To be extractable, the implementation must satisfy certain computability conditions, for example that all quantifiers in conditionals be bounded. For functions, the compiler can choose among several representations: a closure, a dense representation as an array, or a sparse representation as a hash table. The dense representation is unboxed, allowing a cache-efficient contiguous representation of an array of structures and reducing allocation overhead.

**Fig. 5.**
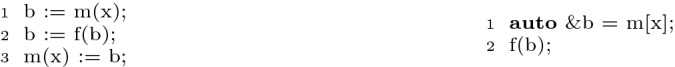
Updating a map in place using the borrow pattern.

Because there are no references in Ivy, there is a risk of copying large structures passed as arguments. However, the lack of aliasing makes it relatively easy for the compiler to detect linear use of data, allowing call and return by reference in the extracted code, and in-place update of structures. Subtype polymorphism in Ivy is implemented by the compiler using smart pointers, allowing structure sharing (and potentially copy-on-write, though this is not yet implemented). In addition, the compiler borrows a technique from the Rust language [22] to introduce references. Consider the Ivy code on the left of Fig. 5 that looks up a value in a map, operates on it, then writes it back into the map. The compiler recognizes this as an instance of the “borrowing” pattern and renders it as the C++ code on the right, which operates on the value in the map by reference. This is possible because the of lack of aliasing and the fact that the compiler understands the underlying data structures. A C++ compiler cannot accomplish this optimization because of the difficulty of pointer analysis in the map implementation and the called operator *f*. Benchmarks of an older Ivy compiler [28] on distributed protocols showed comparable performance to implementation in OCaml and Go, though Ivy is purely value-based, while these languages support references.

*Concurrency.* Although Ivy is a synchronous reactive language, the compiler can extract parameterized distributed programs from Ivy programs in a sound way. In a parameterized module, each action and state variable has a first parameter representing a *location*. The compiler verifies that different locations do not interfere with each-other, and then extracts an executable process that takes its location as a parameter. Ivy guarantees that executing the locations concurrently is observably equivalent sequential execution, based on a left-mover/right-mover argument [15, 28].

*Run-Time Support.* Ivy provide a standard library that includes useful abstractions, such ordered datatypes and arrays, as well as formally specified interfaces to networking services provided by operating systems. In addition, the compiler automatically generates marshaling and unmarshaling code for user-defined datatypes. These facilities make it relatively straightforward to implement verified networked protocols in Ivy.

## Conclusion

Ivy has been designed to provide predictability, stability and transparency in the process of developing verified systems. For this purpose, it integrates a collection of verification techniques that provide these properties, while attempting to maximize the expressiveness of the language, the degree of proof automation, and the efficiency of extracted code. By setting the division of labor between the human and automated provers appropriately, it aims to increase the productivity of the overall process of formal development.
